# Prioritizing Child Health Interventions in Ethiopia: Modeling Impact on Child Mortality, Life Expectancy and Inequality in Age at Death

**DOI:** 10.1371/journal.pone.0041521

**Published:** 2012-08-07

**Authors:** Kristine Husøy Onarheim, Solomon Tessema, Kjell Arne Johansson, Kristiane Tislevoll Eide, Ole Frithjof Norheim, Ingrid Miljeteig

**Affiliations:** 1 Research Group for Global Health, Department of Public Health and Primary Health Care and Center for International Health, University of Bergen, Bergen, Norway; 2 International Center for AIDS Care and Treatment Programs (ICAP) Ethiopia, Addis Ababa, Ethiopia; Tehran University of Medical Sciences, Islamic Republic of Iran

## Abstract

**Background:**

The fourth Millennium Development Goal calls for a two-thirds reduction in under-5 mortality between 1990 and 2015. Under-5 mortality rate is declining, but many countries are still far from achieving the goal. Effective child health interventions that could reduce child mortality exist, but national decision-makers lack contextual information for priority setting in their respective resource-constrained settings. We estimate the potential health impact of increasing coverage of 14 selected health interventions on child mortality in Ethiopia (2011–2015). We also explore the impact on life expectancy and inequality in the age of death (Gini_health_).

**Methods and Findings:**

We used the Lives Saved Tool to estimate potential impact of scaling-up 14 health interventions in Ethiopia (2011–2015). Interventions are scaled-up to 1) government target levels, 2) 90% coverage and 3) 90% coverage of the five interventions with the highest impact. Under-5 mortality rate, neonatal mortality rate and deaths averted are primary outcome measures. We used modified life tables to estimate impact on life expectancy at birth and inequality in the age of death (Gini_health_). Under-5 mortality rate declines from 101.0 in 2011 to 68.8, 42.1 and 56.7 per 1000 live births under these three scenarios. Prioritizing child health would also increase life expectancy at birth from expected 60.5 years in 2015 to 62.5, 64.2 and 63.4 years and reduce inequality in age of death (Gini_health_) substantially from 0.24 to 0.21, 0.18 and 0.19.

**Conclusions:**

The Millennium Development Goal for child health is reachable in Ethiopia. Prioritizing child health would also increase total life expectancy at birth and reduce inequality in age of death substantially (Gini_health_).

## Introduction

The fourth Millennium Development Goal (MDG 4) calls for a two-thirds reduction in deaths of children younger than five years between 1990 and 2015. Fortunately, the under-5 mortality rate (U5MR) is declining in all regions, but many countries are still far from achieving the goal [1], [2]. In Ethiopia, the second most populated country in Africa, the decline in child mortality after 1990 has been steeper than in several other sub-Saharan African countries [3]. Rajaratnam *et al.* estimated a decrease in U5MR in Ethiopia from 201.9 per 1000 live births in 1990 to 101.0 per 1000 live births in 2010 [1]. Still, more than 321,000 children die before they reach five years of age every year [4]. Black *et al.* estimate that neonatal causes (38%), diarrhea (22%) and pneumonia (12%) are the major causes of Ethiopian child deaths [4].

The Ethiopian government and the Federal Ministry of Health have taken positive steps by investing in development and health care. There has been substantial progress on several health, economic and development indicators in Ethiopia, though there is ample room for further improvement, as illustrated by the country’s socioeconomic indicators given in Table 1 [1], [5], [6], [7], [8], [9]. Through their *Plan for Accelerated and Sustained Development to End Poverty (PASDEP)* and *Health Sector Development Program IV (HSDP IV),* the government demonstrates ambitious plans for economic development and health improvements [10], [11].

**Table 1:** Sociodemographic characteristics for Ethiopia (1, 5–9).

Population indicators	
Total population (000)	82825
Population aged under 15	44%
Life expectancy at birth (years)	59.3
**Health indicators**	
Total Fertility Rate	3.9
Maternal Mortality Ratio (per 100,000 live births)	590
Neonatal Mortality Rate (per 1000 live births)	35
Infant Mortality Rate (per 1000 live births)	68.5
Under-5 Mortality Rate (per 1000 live births)	101
One year olds fully immunized against measles	75%
Stunting in children under 5 years of age	47%
HIV prevalence rate	2.1%
Physician per 10,000 population	0.2
**Development indicators**	
Adult literacy rate (>15 years)	29.8%
Gross Domestic Product per capita (PPP $)	934
People living below 1,25 $ a day	39%
Human development index	0.363
Multidimensional Poverty Index	0.562
Income Gini coefficient	29.8
Health expenditure as % of GDP	4.3%
Per capita total expenditure on health (PPP $)	37

Studies on child health show that scale-up of effective interventions could have a high impact on child mortality [12], [13]. But the gap between those in need of care and those who in reality have access to care is large [2], [7], [14]. When the burden of disease is high and there are limited resources to invest in health care, decision makers face difficult dilemmas on where to invest their resources. To make these assessments, decision makers need valid and relevant information concerning the different alternatives and their distributive consequences as well as opportunity costs [15], [16].

However, we lack information on which services will promote rapid health gains and which services to prioritize in a specific country. As of today, models on possible impacts and costs of introducing new interventions and scale-up of interventions exist for larger WHO regions [17], [18], [19], [20], [21], [22]. Contextualized models applying best local evidence give information that is more relevant for decision makers at the country level. Marginal Budgeting for Bottlenecks (MBB) and the Lives Saved Tool (LiST) are new analytic epidemiological tools for policy makers and researchers to evaluate the possible health impacts of scaling-up interventions [23], [24]. Ethiopia is currently using MBB in health planning and prioritizing of health care interventions [10]. Analysis with LiST would give additional information on possible results of scaling-up interventions. Some studies have illustrated how LiST can be used in planning and evaluation of health programs at the regional and country level [17], [25], [26]. However, no studies have done a contextualized LiST analysis in Ethiopia or studied how results from a LiST analysis would impact population health and pure health inequality.

This study aims to estimate the potential health impact of increasing coverage of 14 selected health care interventions targeting child mortality in Ethiopia. We also explore the impact on life expectancy and inequality in the age of death (measured by Gini_health_).

## Methods

In support of evidence-based policy making and priority-setting, we have recently seen a new focus on modeling tools analyzing possible impacts of increased access to health interventions. Rudan and colleagues argue that an optimal priority setting tool “should be able to draw on the best local evidence and guide policy makers and governments to identify, prioritize, and implement evidence-based health interventions for scale-up and delivery” [27]. PopMod [20], Marginal Budgeting for Bottlenecks (MBB) [23] and the Lives Saved Tool (LiST) [24] are current examples of modeling tools which assess the impacts of scaling-up health care interventions using evidence-based data. PopMod and MBB also include costing opportunities. The modeling tools assume that the interventions will be fully implemented. However, actual effects of scale-up depend on factors like human resources and equipment available, health seeking behavior and other context depended factors.

We chose to use LiST, as the most user-friendly and the most recently updated tool available at time of analysis, as we believe these concerns are relevant for policy makers. LiST and MBB is also a part of the new OneHealth tool, which harmonize current modeling tools on costing and impact assessment in the health sector [28]. This paper addresses impacts on health of scaling-up interventions, but does not address costs.

### Lives Saved Tool

We used the Lives Saved Tool (LiST) version 4.43. LiST models changes in maternal and child mortality by scaling-up coverage of health care interventions. The tool operates within the Spectrum model, where maternal and child health data are integrated with demographic (DemProj) and HIV/AIDS (AIM) projections [29]. Evidence-based effectiveness data are from the Child Health Epidemiology Research Group (CHERG), which is an expert group conducting technical reviews on maternal, neonatal and child morbidity and mortality and effectiveness of interventions [30].

### Interventions

We selected 14 essential child health interventions relevant for Ethiopian decision makers through the following process: first, we identified recent recommendations on which interventions to give priority to in resource-constrained settings from the literature [12], [13], [17], [18]. Second, we included interventions that are explicitly considered in Ethiopia, but are not yet implemented, such as the pneumococcal vaccine [31]. The interventions were chosen to reflect the diversity of intervention options and delivery platforms, i.e. according to a) level of care, b) preventive or curative concerns and c) early and late mortality. All interventions target conditions with high burden of disease in Ethiopia [4].

Through meetings with key persons in the Ethiopian Ministry of Health in November 2010 we got access to drafts of the Health Sector and Development Program IV (HSDP IV) and the final draft in August 2011. Access to HSDP IV planning provided information on which interventions were conceived as relevant to the Ethiopian Ministry of Health and also current coverage estimates. This study does not look at the impact of all interventions included in HSDP IV, but the 14 selected child health interventions only. The complete list and description of the 14 interventions are provided in the Appendix S1.

### Modification of LiST Input Data

We modified the newest available LiST projection for Ethiopia (June 2011) by use of contextualized data and evidence. We adjusted life expectancy (UN Population Data) and life table (WHO 2008) in the demographic projection and updated epidemiological and current coverage data in the LiST model (2011) [1], [7], [10]. We used effectiveness data from the Child Health Epidemiology Research Group (CHERG), as we did not have contextualized effectiveness data [30].

**Figure 1:**
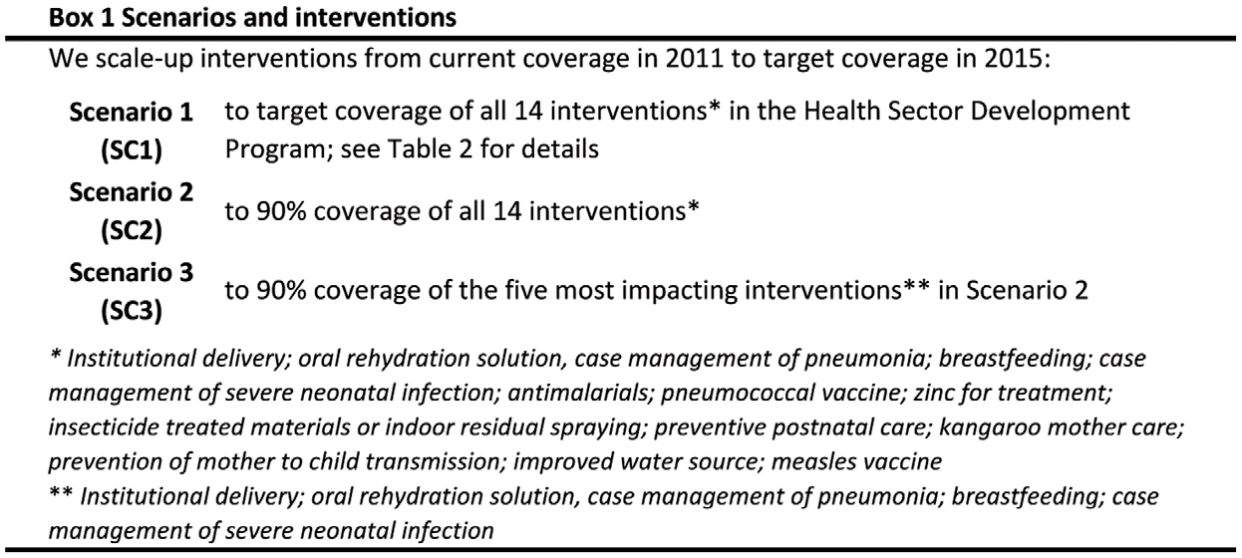
Scenarios and interventions.

We modeled three different scenarios where interventions were scaled-up in one package, as seen in Figure 1. We scaled-up by linear interpolation from current coverage in 2011 to target levels in 2015:

Scenario 1 (SC1) is a scale-up of the 14 interventions to target coverage levels in HSDP IV (base case scenario).
Scenario 2 (SC2) is a scale-up of the 14 interventions to 90% coverage.
Scenario 3 (SC3) is a scale-up of the five most effective interventions identified in scenario 2 to 90% coverage. The nine other interventions are left out of this scenario.

The scenarios include packages of interventions. In the analysis, preventive interventions are scaled-up before treatment interventions scaled-up. The different interventions included will therefore influence each other, and the estimated number of deaths averted by interventions. For example; when pneumococcal vaccine is not included in the package (Scenario 3), LiST estimates that there will be more cases of pneumonia, and therefore also more lives to be averted by case-management of pneumonia (compared to Scenario 2).

Under-5 mortality rate (U5MR), neonatal mortality rate (NMR) and deaths averted are primary outcome measures.

### Life Expectancy and Distribution of Age at Death (Gini_health_) Calculations

Based on the LiST analysis, we estimated two secondary outcome measures: life expectancy at birth and inequality in age at death (Gini_health_). Life expectancy is a long-term outcome and an indicator of overall average population health. Gini_health_ adds information on distribution of age at death around the average.

The LiST analysis provided life tables with data on mortality for the different age groups in a given population, here presented as estimated number of deaths per 100 000. The life tables included impacts on mortality rates among the population below 5 years of age after interventions. We assume that the life tables from LiST represent the average Ethiopian population in 2011 and 2015. In a spreadsheet we included Ethiopian data on fertility and population data from PopMod (2005) to also estimate impact on maternal mortality for scale-up of such interventions, as this is not included in current life tables from LiST [32].

We calculated life expectancy at birth based on the life tables from LiST by use of standard methods [33]. The calculations are simplified, as the life tables are non-dynamic. The life tables were then used to estimate inequality in age at death (Gini_health_) by a method first suggested by LeGrand [34], later developed by others [35], [36], [37]. Gini_health_ is here used as a measure of overall health inequality in a population [34], [36], [38]. In the literature, the Gini index is commonly used to analyze income distribution within populations [39]. Wagstaff and others have applied the Gini index to analyze distribution of health within populations. Gini_health_ hereby describes the degree of overall health inequality, defined as inequality in age at death in a given population [35]. We calculated Gini_health_ by use of equation 1 [37]:


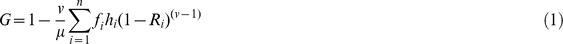


Where *μ* is the average health in this population, *h*_i_ is age of death, *f*_i_ is the sample proportion in the *i*th group, and *R_i_* is the rank of this group (rank 1 is the rank of the best-off group with highest age of death). The parameter *v* is a parameter reflecting aversion to inequality that we have set to *v* = 2 in this study (the value used in the standard Gini) [35]. The Gini coefficient equals to zero for perfect equality and one for the most unequal distribution. Gini_health_ normally varies between 0.10 and 0.50 [36]. Socioeconomic inequality in health is an important contributor to overall inequality in health, and can be measured within a population ranked by wealth by the analogous Concentration Index [35]. This is however not done in this paper, where we study pure health inequality, by differences in age at death, not by differences in wealth.

## Results

Increasing coverage of the 14 interventions to target levels in the Ethiopian HSDP IV could avert 114,600 child deaths by 2015 (Table 2).

**Table 2:** Estimated deaths averted from scaling-up health care interventions in Ethiopia from 2011 to 2015.

Intervention	Current coverage(2011)	Scenario 1	Deaths avertedScenario 1	Scenario 2	Deaths avertedScenario 2	Scenario 3	Deaths avertedScenario 3
Institutional delivery	15.7%	65.0%	26700	90.0%	45900	90.0%	45900
*Labor and delivery management* *	*3.1%*	*45.5%*	*11800*	*90.0%*	*21500*	*90.0%*	*21500*
Oral Rehydration Solution	37.0%	65.0%	26700	90.0%	42600	90.0%	52800
Case management of pneumonia	0.0%	17.0%	4200	90.0%	20800	90.0%	27000
Breastfeeding	49.0%	57.0%	2800	90.0%	17600	90.0%	18500
Case management of severe neonatal infection	25.0%	42.0%	5800	90.0%	15700	90.0%	20200
Antimalarials	8.0%	54.0%	8900	90.0%	12600	8.0%	0
Pneumococcal vaccine	0.0%	90.0%	12600	90.0%	12500	0.0%	0
Zinc for treatment	0.0%	62.0%	9900	90.0%	12000	0.0%	0
Insecticide treated materials or indoor residual spraying	42.0%	65.0%	4200	90.0%	8900	42.0%	0
Preventive postnatal care	5.0%	25.0%	2300	90.0%	8700	5.0%	0
Kangaroo mother care	6.3%	45.5%	4800	90.0%	8200	6.3%	0
Prevention of Mother To Child Transmission ofHIV (PMTCT)	8.0%	76.0%	5700	90.0%	7200	8.0%	0
Improved water source	65.2%	98.3%	5800	90.0%	4400	90.0%	0
Measles vaccine	77.0%	90.0%	100	90.0%	100	77.0%	0
**Total**			**114600**		**217200**		**164400**

numbers of lives averted by teves averted by teh breastfeeding intervention increases and the number of lives averted by other.

We model scale-up in three scenarios. Current coverage data (2011) are from HSDP IV and the Ethiopian Demographic and Health Survey (2005). The definitions of the interventions can be accessed through Appendix S1 and details about scenarios 1, 2 and 3 can be found in Figure 1.

* Labor and delivery management is a subcomponent of the institutional delivery intervention.

By increasing coverage to 90% of all 14 interventions, an additional 102,600 deaths could be averted (217,200 deaths averted in total). The five most effective interventions are: 1) institutional delivery, 2) oral rehydration solutions (ORS), 3) case management of pneumonia, 4) breastfeeding and 5) case management of severe neonatal infections. Together, these interventions account for 57.8% and 65.7% of the deaths averted in Scenario 1 (SC1) and Scenario 2 (SC2), respectively, and all deaths averted in Scenario 3 (SC3) (66,200, 142,600 and 164,400 averted deaths, respectively).

**Figure 2:**
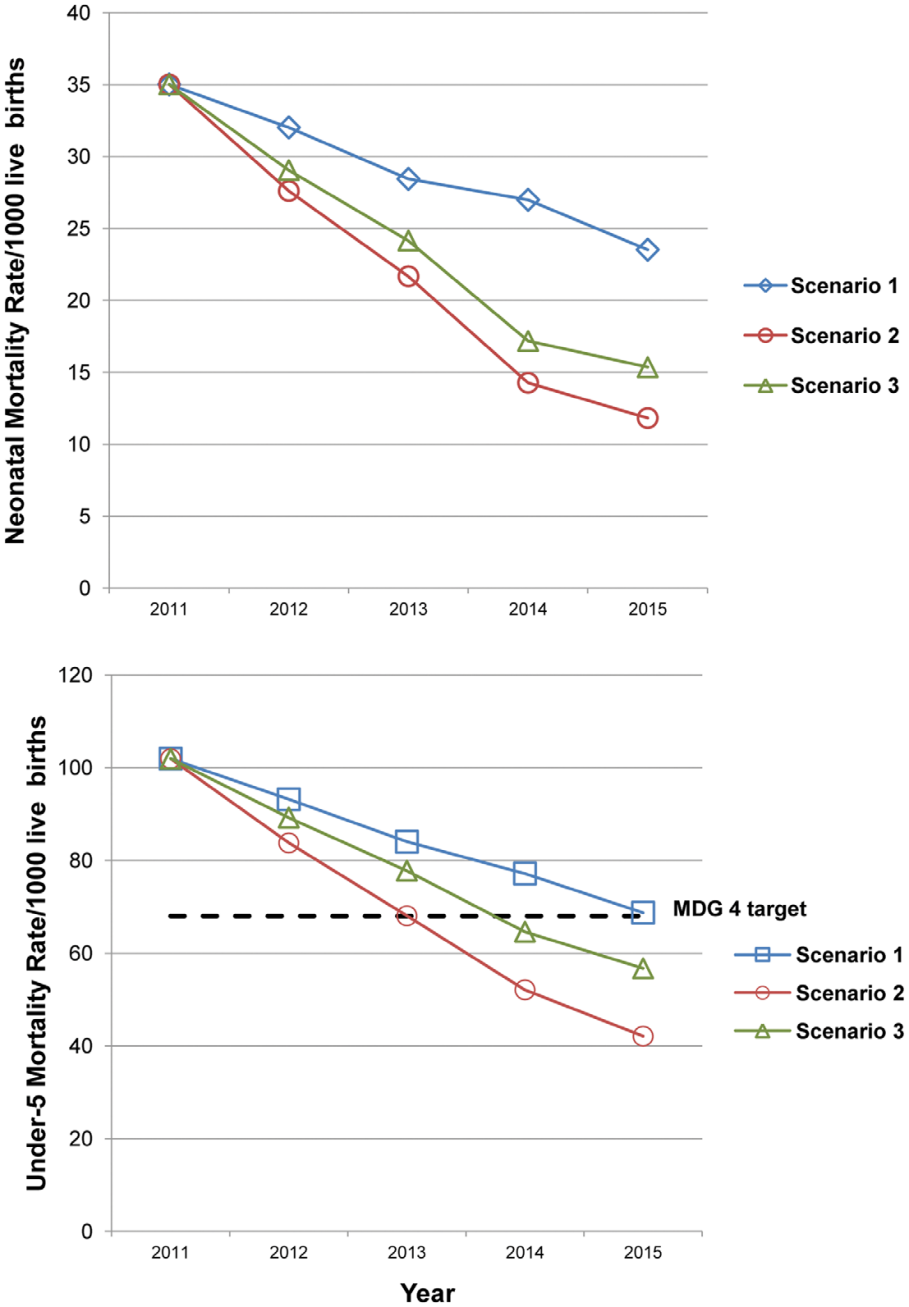
Impacts on neonatal mortality rate and under-5 mortality rate of scaling-up interventions in Ethiopia. Scenario 1 is in blue (with squares), scenario 2 in red (with circles) and scenario 3 in green (with triangles). See Figure 1 and Appendix S1 for details about the different scenarios and interventions. The dotted line shows the Millennium Development Goal 4 (MDG 4) target (under-5 mortality rate <68/1000 live births).

Figure 2 shows how scaling-up coverage of interventions can impact mortality rates. A decline in neonatal mortality rate (NMR) from 39.0 in 2010 to 23.5, 11.8 and 15.4 per 1000 live births is expected if coverage levels are increased according to target levels in SC1, SC2 and SC3 by 2015. This is a 32.8%, 66.2% and 56.1% reduction compared to the current neonatal mortality rate (39/1000 births). Implementation of all 14 interventions in line with SC1, SC2 and SC3 by 2015 yield an under-5 mortality rate (U5MR) of 68.8, 42.1 and 56.7 per 1000 live births, respectively. Compared to the current U5MR, the reduction is then 32.6%, 58.7% and 44.4%. Ethiopia can therefore reach MDG 4 within a period of 5 years, and with only five interventions arrive at an U5MR of 56.7 per 1000 live births if SC3 is implemented.

**Figure 3:**
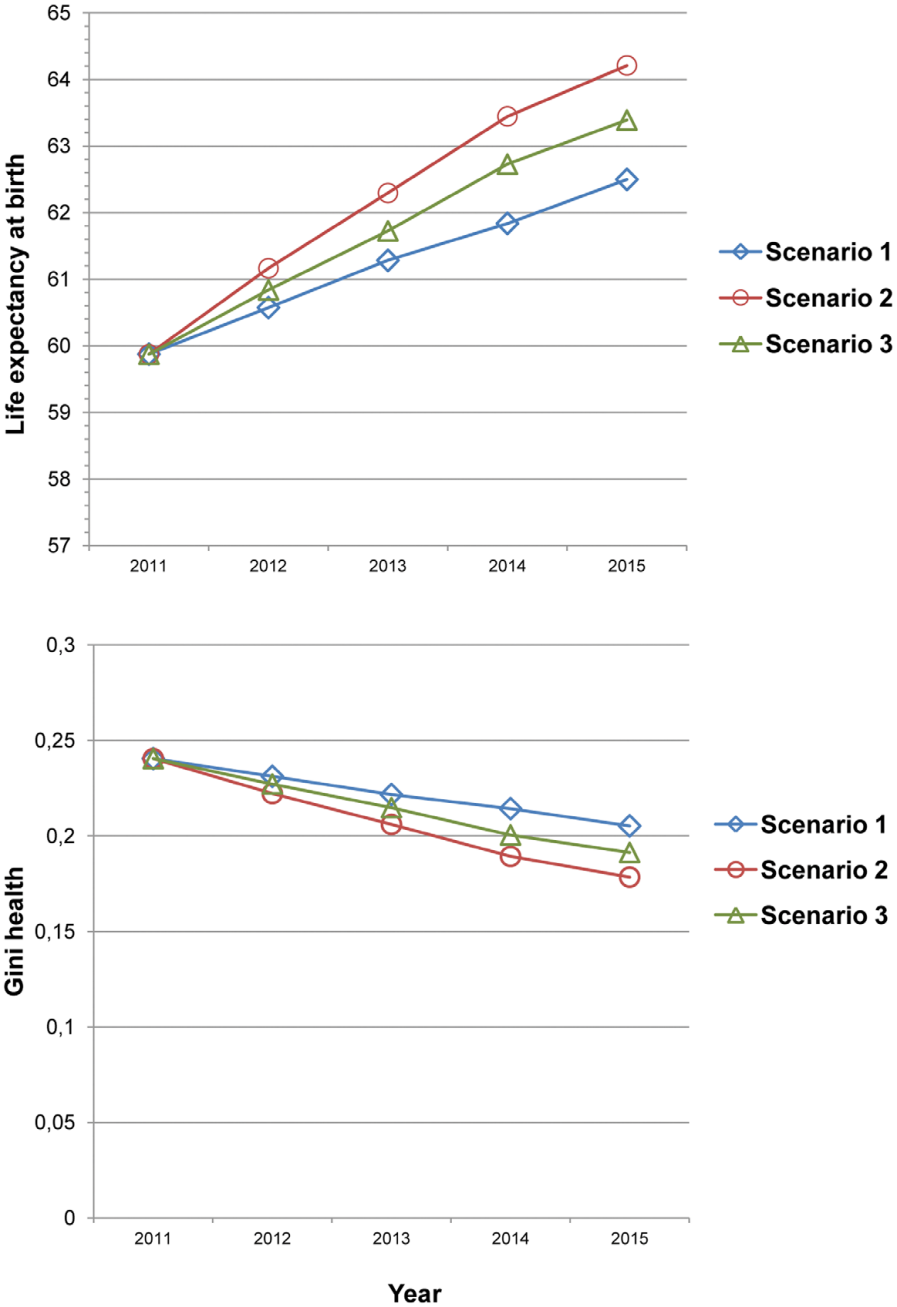
Temporal impacts on life expectancy and Gini_health_ of scaling-up interventions between. Scenario 1 is in blue (with squares), scenario 2 in red (with circles) and scenario 3 in green (with triangles). See Figure 1 and Appendix S1 for details about the different scenarios and interventions.

SC1, SC2 and SC3 yield a large increase in life expectancy and reduced inequality in age at death (Gini_health_) (Figure 3). Life expectancy at birth increases to 62.5 (+2.6), 64.2 (+4.3) and 63.4 (+3.5) by increasing coverage of interventions according to SC1, SC2 and SC3, respectively. This corresponds to a 4.4%, 7.2% and 5.9% increase in life expectancy at birth. Without scale-up of interventions, Gini_health_ in Ethiopia is estimated to be 0.24 in 2015. Scaling-up to SC1, SC2 and SC3 levels would lead to a reduction in inequality in age at death (Gini_health_) at 0.21 (−0.03), 0.18 (−0.06) and 0.19 (−0.05), or a reduction from 2011 by 14.7%, 25.8% and 20.4%, respectively.

## Discussion

Our LiST analysis shows that scaling-up selected child health interventions can have great impact on mortality, life expectancy and inequality in age at death. Even implementation of a package of only five interventions is estimated to yield an U5MR of 56.7 per 1000 live births. The MDG 4 target of an U5MR of 68 per 1000 live births is achievable. Our estimates provide support for giving priority to child health interventions in Ethiopia also in a broader perspective. An increase in life expectancy after reduction in premature mortality could be expected, and here the extent of the increase in life expectancy is noteworthy. It is also important to note that prioritizing child health will have great impacts towards a more equal distribution of health in the population, as seen in the substantial reduction in Gini_health_.

### Which Interventions to Prioritize?

Given budget constraints, policy makers in Ethiopia must deal with the tragic trade-off: should they opt for a package of a few very effective interventions at a high coverage rate, a large package at a medium coverage rate, or a mix of these packages? And how much weight should be given to efficiency and equity versus other concerns like affordability or donor preferences?

If the policy makers opt for a few very effective interventions at a high coverage rate, our results indicate that a package of institutional delivery, oral rehydration solution (ORS), case management of pneumonia, breastfeeding and case management of severe neonatal infection could have considerable impact on child survival. These five interventions differ in their level of care, and implementation of them demands various competencies and strategies to succeed. More than 80% of the Ethiopian population lives in rural areas, and our analysis shows that scaling-up outreach services in these settings could avert many deaths. Case management of pneumonia and ORS could alone avert 30,900, 63,400 or 79,800 deaths if scaled-up by 2015 (SC1, SC2 and SC3), and are simple interventions that can be delivered in community outreach programs [17]. HSDP IV aims to decentralize the health system and focuses on preventive and promotive components [10]. Although outreach services are not yet fully implemented, it still might be more feasible than scaling-up more comprehensive services, i.e. institutional delivery and management of severe neonatal infections. Also, community-based interventions have shown to be more equitable distributed than interventions given at health facilities [40]. On the other hand, as others have shown, inclusion of clinical care at the facility level is necessary to reduce newborn as well as maternal deaths [13], [41]. The many possible deaths averted from institutional delivery and management of neonatal infections in our study supports investment also in these clinical interventions. A high-impact intervention such as institutional delivery will save five times more lives than insecticide-treated materials or indoor residual spraying intervention, but will also require more investment in terms of resources and funding than typical “quick-fix” solutions. Although policymakers might prioritize more comprehensive interventions when aiming at averting most deaths, other factors like lack of health workers, people’s preferences or donor-driven priorities might force decision makers to compromise their overall strategy. Contextualized knowledge at country level is needed to ensure that the right interventions are prioritized [17]. They should be supplemented by costing data in support of maximization of the resources available. Numerous studies also indicate that intervention scale-up needs to be accompanied by a general health system strengthening [17], [42].

### The Effects of Different Coverage Rates

Interventions affect mortality depending on the effectiveness of the interventions, the risk of the condition(s) and the extent of scale-up. In general, higher increases in coverage will save more lives. There are striking gaps between current coverage and the target levels in the scenarios we modeled. In SC1, with coverage targets from the HSDP IV, the extent of scale-up differs among the 14 interventions. SC1 is probably the most realistic scenario for a resource-constrained country such as Ethiopia. According to the Ministry of Health’s estimates, MDG 4 should be achievable if all 70 interventions in HSDP IV are scaled-up [10]. In the literature, the importance of achieving universal coverage and access to health care services for all has received increased attention [16], [43]. SC2 and SC3 present alternative examples of how to achieve near universal coverage for a smaller package of essential services would have high impact on life expectancy and inequality. However, Ethiopia is far from achieving universal health coverage of essential child health interventions. Scaling-up health interventions to 90% remains a long-term goal and will require improved quality and adequate human and financial resources for successful implementation.

The Ethiopian government has demonstrated ambitious plans for economic development and health improvements, with the goal that every section of the population should be reached by basic health interventions by 2015 [10], [11]. For instance, through HSDP IV, the government aims to increase coverage of institutional delivery from 15.3% to 65.0% (2011–2015). Over the past years, 253 new health centers have been built and 1,457 health stations have been upgraded to health centers to enable these facilities to perform emergency obstetric and neonatal care services [10]. This illustrates that institutional delivery is a priority for the Ministry of Health, and our study shows that an increase in coverage could save 26,700 lives if they reach their target (Table 2, SC1).

The Ministry of Health has chosen a policy with multiple interventions and more modest increases in coverage. Nevertheless, the targets of this policy will be challenging to carry out during the short period of time (2011–2015). Although they have shown advanced planning through the development of the HSDP IV and mortality rates are declining, previous targets have not always been met, and declines in mortality differ between regions and types of health services [7]. Previous studies have noted that there is not only a coverage gap, but also a gap in quality of care and socioeconomic inequity in access to health services in sub-Saharan Africa [14], [44], [45]. Therefore, Ethiopian policymakers should also pay attention to equity, quality and sustainability when scaling-up interventions.

### Extending the Results: Adding Life Expectancy and Gini_health_

Within the literature of disease control priorities, much attention has been given to lives saved and mortality reductions, as well as cost-effectiveness of interventions [21]. There has been less discussion of reducing inequality in age at death [46]. When aiming to improve health, we need to discuss how to measure health outcomes and how different measurements should be included to secure a thorough evaluation of the impacts of scale-ups. Our study explores several health outcomes (deaths averted, mortality rates, life expectancy and Gini_health_) and thereby provides useful information for policy makers. Figure 3 illustrates the impacts of scale-up on life expectancy and Gini_health_. We believe it is relevant to look at overall health outcomes in addition to child mortality rates alone.

A low life expectancy indicates that the population faces many potential risks that increase the chance of premature death. Life expectancy includes a lifetime perspective rather than a snapshot of mortality rates for different age groups. Our analysis shows that if fewer children (and their mothers) die prematurely, life expectancy would increase substantially. Further, our analysis shows that an investment in child health will reduce inequality in age at death by up to 25.8% (SC 2). By studying Gini_health_, we get information about the impacts on the overall distribution of health within the population. Our empirical analysis suggests that an improvement in child health would both increase life expectancy and reduce inequality in age at death, as there are many potential life-years lost when large parts of the population die prematurely. In real life, the reality is that policy makers consider priorities between interventions aimed at different age groups. As Robberstad and Norheim discuss, inclusion of life expectancy and Gini_health_ would be valuable for priority comparisons between childhood interventions and e.g. non-communicable diseases in adults [38]. LiST, as of 2012, provides information on the possible impacts by intervention and age group (neonatal, infant and under-5), but inclusion of a broader range of intervention types and age groups (including adult health) would be preferable from a comprehensive policy perspective.

Decision making should draw on the best possible estimates available, and different outcome measurements should be used to ensure that a variety of concerns are given due attention [47], [48].

### Strengths and Limitations of the Study

Our study is the first to use LiST in an Ethiopian policy context, and LiST results have not been used in population health impact analysis previously. We found that LiST is a useful tool that integrates epidemiological and effectiveness data and can be used to model impact of scaling-up interventions. The epidemiology, coverage, and health status data we used are from most recent estimates. Most of the data are estimates or secondary data and interpretations of our results should take into account the uncertainty of the data used. Previous validations of LiST have shown that LiST estimates most often match measured mortality, which gives us reason to trust the effectiveness data from LiST [25], [49]. On the other hand, some have argued that use of clinical effectiveness data might overestimate deaths averted [50].

Our results should be interpreted carefully, as our study does not say anything about services not included in the analysis. Also, the LiST analysis does not express how the interventions influence quality of life.

We assumed that implementation would be fully realized. In reality, the effect of the scale-up will be affected by quality of care, accessibility and utilization of the services. Overcoming these barriers would imply large costs in scale-up of coverage rates. Although the cost-effectiveness of the different interventions varies, all interventions in our study have earlier been shown to be very cost-effective [21]. The Ethiopian Ministry of Health estimates that an additional $12 USD per capita per year is required to scale-up 70 health interventions to base-case scenario targets [10]. An additional contextualized cost-effectiveness study, exploring the costs of scale-up of interventions, would be a valuable supplement to our study. Others have described that scaling-up interventions to high coverage rates will be more challenging and require additional costs [51]. Reaching rural parts of the Ethiopian population without existing health care services will require additional resources, and calculating the possible costs of this process is an area for further investigation.

If all interventions were scaled-up, there would probably be other beneficial impacts on health (due to availability of health workers, better health facilities etc.), and our estimates might underestimate the potential decreases in mortality compared to a real-life scale-up. Also, we did not assess non-health benefits (such as effect on education and future income) for child interventions, which in general are known to be substantial [52].

### Conclusions

Our study estimates that increasing coverage of 14 selected health care interventions in Ethiopia would reduce child mortality substantially, increase life expectancy and reduce inequality in health (Gini_health_). Both the choice of interventions and the extent of scale-up will have consequences for the number of deaths averted. LiST analysis provides additional information applicable in priority setting by making the outcomes of alternative scenarios more explicit. Various concerns are important when making health policies. Evaluation of available options is inevitable, and should be based upon best available evidence.

## Supporting Information

Appendix S1 **Description of interventions.**
(DOC)
