# Supplementary material for: Prioritizing Child Health Interventions in Ethiopia: Modeling Impact on Child Mortality, Life Expectancy and Inequality in Age at Death
Source: PLoS One. 2012 Aug 7;7(8):e41521. doi: 10.1371/journal.pone.0041521 (PMC3413690; doi:10.1371/journal.pone.0041521)
Supplement: Appendix S1 — Description of interventions. (DOC) [file pone.0041521.s001.doc]

#### Appendix S1

Description of interventions

|  | **Intervention** | **Description of intervention** |  |
| --- | --- | --- | --- |
|  | **Institutional delivery** | Births within a facility, skilled delivery care |  |
|  | Clean birth practices | Hand washing before delivery, cleaning the perineum before birth, using a clean birth surface, clean cord cutting. |  |
|  | Immediate assessment and stimulation | Immediate newborn assessment and stimulation, including drying of baby. |  |
|  | Labor and delivery management | Deliveries with access to a specific level of care (at facility level) if needed. |  |
|  | Essential care for all women and immediate essential newborn care | Skilled birth attendants monitor labor progress with a partograph. Detection of complications, infection control and clean delivery. Episiotomy can be done if needed. Assuring adequate temperature control and initiation of breastfeeding of the baby. |  |
|  | Basic Emergency Obstetric Care | Initial management of delivery at a health center and case management of direct obstetric complications (preeclampsia/eclampsia treatment, antepartum hemorrhage, postpartum hemorrhage, prolonged/obstructed labor, abortion, ectopic pregnancy and severe infections). Shock management, MgSO4, pain relief, ABC, parenteral antibiotics, parenteral oxytocics, IV fluids, instrumental delivery, manual removal of the placenta and retained products. |  |
|  | Comprehensive emergency obstetric care | Hospital based management of delivery and case management of direct obstetric complications (preeclampsia/eclampsia treatment, antepartum hemorrhage, postpartum hemorrhage, prolonged/obstructed labor, abortion, ectopic pregnancy and severe infections). Includes services in Basic and emergency obstetric care (BemOC), but also ultrasound, culdocentesis, induction, laparotomy, salpingectomy, blood transfusion, caesarian section, hysterectomy, symphysiotomy, balloon tamponade, uterine ligature, surgical infection control, episiotomy. |  |
|  | Neonatal resuscitation | Detection and resuscitation with bag and mask within health facility. |  |
|  | Antibiotics for preterm premature rupture of membranes (MgSO4) | Administration of oral erythromycin to women with premature rupture of membranes who are not in labor (250 mg orally x 4 for 7 days). |  |
|  | Active management of the third stage of labor | Administration of prophylactic oxytocics. Early cord clamping and cutting. Controlled cord traction/massage to deliver the placenta. |  |
|  | Induction of labor for pregnancies lasting 41+ weeks | Percent of women who are 41 or more weeks pregnant who are managed with induction of labor if needed. |  |
|  | **Preventive postnatal care** | Visit/contact within two days of birth: hygienic cord care, skin hygiene, temperature control, early detection of illnesses and extra care of all low birth weight babies. |  |
|  | **Kangaroo mother care** | Skin-to-skin contact between a mother and newborn, frequent and exclusive breastfeeding and early discharge from the hospital. Must be given in facility. |  |
|  | **Case management of severe infection in neonates** | 1) Injectable antibiotic case management of severe infection in neonates (50% scale-up). 2) Hospitalized care including oxygen therapy, IV antibiotics, IV fluids, blood transfusion, phototherapy and other supportive management (50% scale-up). |  |
|  | **Prevention of mother-to-child transmission of HIV/AIDS (PMTCT)** | % of HIV-infected pregnant women who received antiretroviral drugs for PMTCT in the last 12 months out of the estimated % of HIV-infected pregnant women. HAART. |  |
|  | **Breastfeeding** | Breastfeeding behavior (exclusive, predominant, partial or not breastfeeding). In this study we look at coverage of breastfeeding prevalence <1 month and between 1 to 5 months. |  |
|  | **Case management of pneumonia** | Children (1–59 months) with suspected pneumonia or ARI treated with oral antibiotics. |  |
|  | **Improved water source** | Homes with improved water source. |  |
|  | **Insecticide-treated materials or indoor residual spraying** | Households with at least one insecticide treated net or covered by indoor residual spraying. |  |
|  | **Zinc for treatment** | Children (0–59 months) with diarrhea receiving zinc supplementation (20 mg zinc daily for 14 days). |  |
|  | **Oral rehydration solutions (ORS)** | Children (0–59 months) with diarrhea given ORS (sachets, pre-mixed solutions of ORS, homemade sugar-salt solutions or recommended home fluids). |  |
|  | **Antimalarials** | Children (1–59 months) with a fever receiving any appropriate anti-malarial. |  |
|  | **Measles vaccine** | Infants having received two doses of measles containing vaccine (MCV). |  |
|  | **Pneumococcal vaccine** | Infants having received three doses of pneumococcal vaccine. |  |
